# In Vitro Propagation and Acclimatization of Banana Plants: Antioxidant Enzymes, Chemical Assessments and Genetic Stability of Regenerates as a Response to Copper Sulphate

**DOI:** 10.3390/plants10091853

**Published:** 2021-09-07

**Authors:** Doaa M. Abou Elyazid, Abdel-Moety Salama, Abdel Fattah M. El Zanaty, Neama Abdalla

**Affiliations:** 1 Horticulture Department, Faculty of Agriculture, Kafrelsheikh University, Kafr El-Sheikh 33516, Egypt; 2 Physiology and Breeding of Horticultural Crops Lab, Horticulture Department, Kafrelsheikh University, Kafr El-Sheikh 33516, Egypt; 3 Genetics Department, Faculty of Agriculture, Menoufia University, Al Minufya 32514, Egypt; zanaty1966@gmail.com; 4 Plant Biotechnology Department, Genetic Engineering and Biotechnology Research Division, National Research Centre, 33 El Buhouth St., Dokki, Giza 12622, Egypt; neama_ncr@yahoo.com

**Keywords:** endophytic, *Musa* sp., catalase, peroxidase, RAPD, electrolyte leakage

## Abstract

Developing a successful protocol for banana in vitro culture is a guarantee for the mass propagation of pathogen-free, high-quality, true-to-type planting materials with low production costs. The current work aimed to investigate the influence of increasing copper levels in an MS medium on endophytic bacterial contamination; shoot multiplication; rooting and the acclimatization of in vitro cultured banana; minerals and chlorophyll content; antioxidant enzymes activity; electrolyte leakage; and the genetic stability of banana regenerants. Four different concentrations of copper sulphate (0.025 as a control, and 30, 60, and 120 mg L^−1^) were examined. The growth of the endophytic bacteria was inhibited at 60 mg L^−1^ of copper sulphate which recorded zero contamination, without a significant difference at 120 mg L^−1^. However, 0.025 mg L^−1^ of copper sulphate was optimal for the maximum shoot number and shoot length (10 shoots and 6 cm, respectively) without significant differences at 30 mg L^−1^. The root length of banana plantlets was significantly enhanced at 30 mg L^−1^ of copper sulphate but without significant differences to the control, regarding the number of roots (9.92 cm and 3.80 roots, respectively). In vitro plants were acclimatized successfully at 30 mg L^−1^ of copper sulphate with 100% survival. The uptake of minerals, antioxidant enzyme activity and electrolyte leakage was improved because of the copper sulphate, but the chlorophyll level decreased. RAPD profiling showed polymorphism in only one plant treated with 60 mg L^−1^ of copper sulphate, with an average of 1.8%. The genome template stability percentage was almost 100% for all treated plants.

## 1. Introduction

Banana (*Musa* sp.), which belongs to the family *Musaceae*, is considered one of the most popular fresh fruits worldwide and all cultivars of banana are nearly derived from *Musa balbisiana* and *Musa acuminata* [1]. Its name originates from the Arabic word “banan,” meaning the finger. Banana has a high nutritive value; a high content of pyridoxine: vitamin B6, potassium, carbohydrates and fiber. Banana plants are commercially propagated through the tissue culture technique [2], which can provide mass propagation, rejuvenation of older varieties, disease elimination, conservation of genetic resources, and the management of abiotic and biotic stresses [3,4]. Microbial contamination is one of the most important obstacles, as it prevents the successful micropropagation of in vitro plants [5]. Therefore, several methods have been used to obtain aseptic cultures of explants, such as ultraviolet, autoclaving of media [6] or the use of chemical antiseptic agents like sodium hypochlorite and mercuric chloride, whereas, antibiotics and copper sulphate can be used for eliminating the endogenous contamination [5].

As an essential micronutrient for plant growth, copper (Cu) is needed for several physiological functions, including photosynthesis, respiration, oxidative stress response, electron transport, plant hormone signaling, and cell wall biosynthesis and its lignification [7]. As a cofactor in many enzymes, Cu has a crucial role in many plant enzymes such as amino oxidase, laccase, superoxide dismutase (SOD), and polyphenol oxidase [5]. Copper and its compounds are important ingredients in several antibacterial and antifungal compounds, which have been applied against a wide range of pathogens including viruses, fungi and bacteria [8,9]. However, Cu is too toxic at high concentrations causing a reduced photosynthetic rate, increasing the formation of reactive oxygen species (ROS), causing rising leakage of potassium ions from the roots [10], and inducing the deficiency of iron [5]. Many reports have indicated the positive effects of higher Cu concentrations on in vitro culture of many plants, such as barley [11], tobacco [12], *Lepidium sativum* [13], rice [14], carrot [15] and *Gymnema sylvestre* [16], as reported by [5,6].

Few studies have reported the phytotoxicity of copper [17,18] on *Musa acuminata* and on *Philodendron selloum* [5] plants, which confirmed that the high activity of the antioxidant enzymes could cause ROS detoxification, hence, decreasing the oxidative damage of plants. Many studies have been published about the different stresses on in vitro *Musa* sp., such as water stress [19], osmotic stress [20], cold stress [21], stress of nanoparticles [22,23,24], and salinity stress [4], but very few on copper stress [18,25].

Optimizing the concentration of CuSO_4_ in banana tissue culture media could be a more efficient way to improve the growth and development of micropropagated banana plants by enhancing shooting, rooting [25] and subsequent acclimatization. Therefore, the current study aims to evaluate the impact of increasing the concentration of Cu in the medium by the supplementation of the MS medium [26] with different high concentrations of copper sulphate; on endophytic bacterial contamination, shooting, rooting and acclimatization of in vitro cultured banana; chemical contents; antioxidant enzyme activity; electrolyte leakage; genetic fidelity and the stability of banana regenerants with the target of in vitro mass propagation of high-quality banana plants for commercial production.

## 2. Results and Discussion

### 2.1. Copper Sulphate and Endophytic Bacterial Contamination Percentage

**Figure 1:**
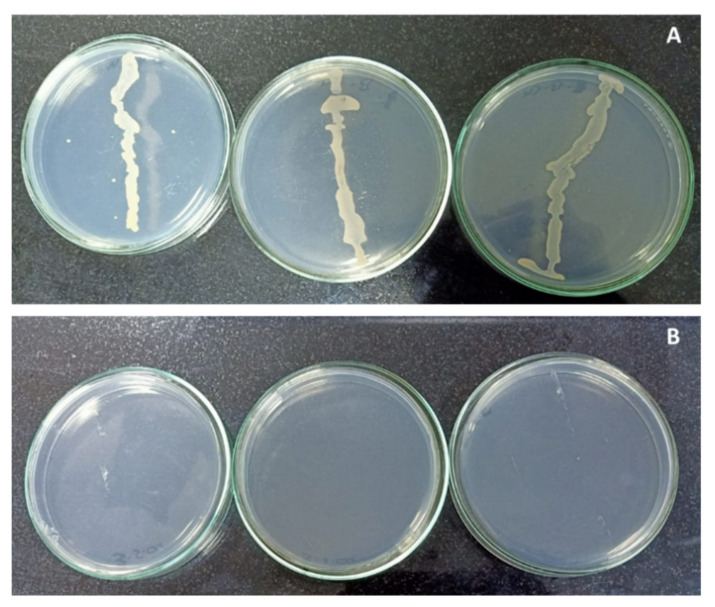
Effect of copper sulphate on endophytic bacteria contamination. Photo (**A**): Control (0.025 mg L^−1^) showed bacterial contamination. Photo (**B**): 120 mg L^−1^ was free from contamination.

The primary experiment, in Figure 1, showed that copper sulphate had a significant negative effect on the growth of isolated endophytic bacteria, which was completely inhibited at the higher concentration of copper sulphate (120 mg L^−1^), as it recorded zero contamination. Whereas, 0.025 mg L^−1^ of copper sulphate, which was found in the MS medium at this lower concentration, reported bacterial contamination. Copper exhibits antimicrobial activity, making it one of the most important components in most effective bactericides [5,8,27,28,29]. Accordingly, copper sulphate could be applied over other chemicals for controlling and eliminating the bacterial contamination. Depending on the primary experiment and according to the obtained information from the literature, four different concentrations of copper sulphate (0.025, 30, 60 and 120 mg L^−1^) were selected to limit the bacterial contamination percentage in banana in vitro cultures.

The recorded results also clearly indicated that the increasing of copper sulphate concentration highly decreased the contamination percentage. The best result was observed with 60 mg L^−1^ of copper sulphate treatment. No significant differences were observed with 120 mg L^−1^, where zero contamination was recorded (Table 1).

The recorded results agree with the findings of the study carried out by [5] on *Philodendron selloum*, in which the fortification of copper sulphate in the two concentrations (70 and 140 mg L^−1^) to the culture medium, completely inhibited the growth of endophytic bacteria compared to the control which recorded 100% contamination.

### 2.2. Copper Sulphate and Shoot Multiplication

In general, there were significant differences among copper sulphate treatments on all recorded measurements except in the number of leaves/shoot where the differences were insignificant. The highest value of shoots (11) was recorded with 30 mg L^−1^ of copper sulphate treatment followed by the control (10), without significant differences between them. Moreover, the number of shoots, as well as shoot length, decreased to almost half by increasing copper sulphate concentration. The control treatment (MS medium copper level) achieved the maximum shoot length (6 cm) without significant differences with 30 mg L^−1^ of copper sulphate (Table 1).

The current study revealed that the fortification of the multiplication medium with 30 mg L^−1^ of copper sulphate had no significant effect on banana shooting compared to the control treatment. The obtained results differ from those recorded by [5] where, copper sulphate at 35 mg L^−1^ was the best treatment regarding the number of shoots in *Philodendron selloum*, with significant differences with the other higher concentrations in this study. The optimum concentration of copper in the culture medium mainly depended on the plant species [30]. In terms of shoot multiplication, some plants responded significantly to lower concentrations of Cu (0.01 to 20 mg L^−1^) [6,31,32]. Whereas, others, such as Arundo, can tolerate copper sulphate up to 300 mg L^−1^ without any adverse effects on its growth and production [5].

On the other hand, according to the current study, increasing the copper sulphate concentration to 60 and 120 mg L^−1^ yielded the opposite effect on the number of shoots. Similar results were observed by some previous researchers that reported a significant reduction in the number of shoots under high copper concentrations [5,33]. The higher copper levels reduced the uptake and transport of some essential metallic elements, such as iron and zinc, that resulted in a negative effect on shoot multiplication [34]. The toxic and useful copper content in the medium depended, to a great extent, on the plant species. Thus, the appropriate copper level should be accurately estimated [6].

### 2.3. Copper Sulphate and In Vitro Rooting

The results shown in Table 2, clearly demonstrated that the (30 mg L^−1^) copper sulphate treatment registered a high efficiency for enhancing the in vitro rooting and growth development of banana plantlets. The highest significant values (14.26 cm, 2.47 g and 9.92 cm) were observed at 30 mg L^−1^ of copper sulphate for plantlet length, fresh weight, and root length, respectively, compared to the control, while there were no significant differences in the number of leaves or the number of roots. Previous investigations reported that the rooting of micropropagated banana plantlets was stimulated by supplementing 4 mg L^−1^ of copper sulphate into a rooting medium, while the rooting was reduced in a medium fortified by 8 mg L^−1^ of copper sulphate [25]. It could be concluded that the better responses of banana in vitro cultures for some growth parameters at higher concentrations of Cu, as compared to the control, were due to the fact that Cu could reduce the endogenous bacterial contamination of explants.

### 2.4. Copper Sulphate and Acclimatization

**Figure 2:**
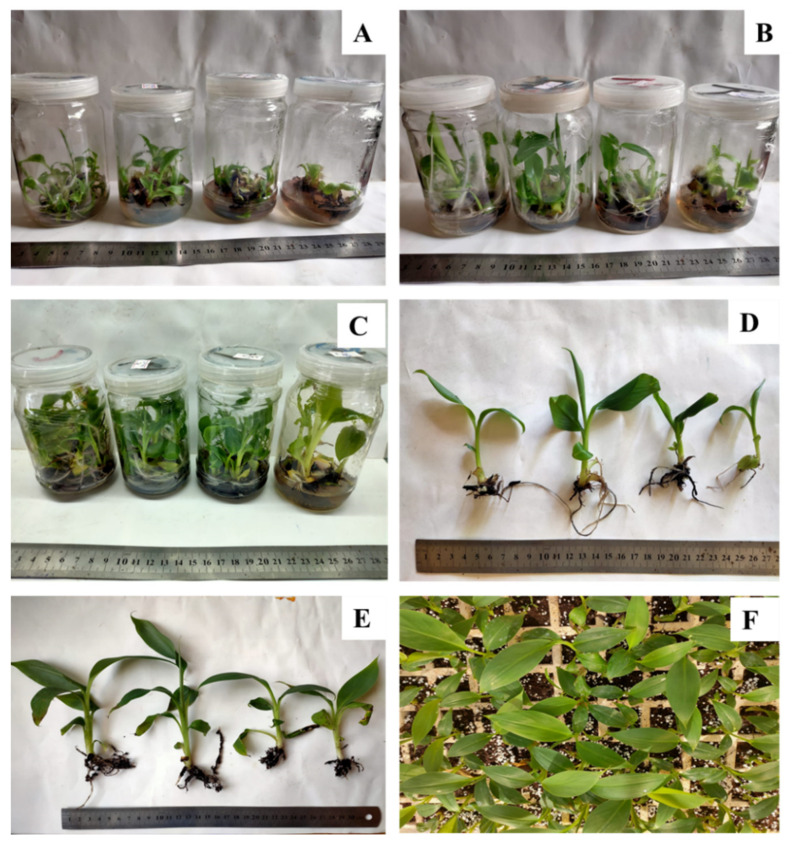
Effect of copper sulphate on in vitro propagation and the acclimatization of banana plants. (**A**) Shoot multiplication in MS medium supplemented with 3 mg L^−1^ BA + 1 mg L kin^−1^. (**B**) Plantlets in elongation medium (MS +1 mg L^−1^ BA). (**C**) Plantlets in rooting medium (MS + 1 mg L^−1^ IBA). (**D**) In vitro rooted plants. (**E**,**F**) Acclimatized plants after 4 weeks (Control, 0.025 mg L^−1^ at Left and 120 mg L^−1^ copper sulphate at right).

The obtained results in Table 3 and Figure 2 showed the effect of copper sulphate on the survival and growth parameters of acclimatized banana plants. The optimum treatment for plant length, root length, leaf area and fresh weight was 30 mg L^−1^ with significant differences observed with the other treatments. Despite this, Cu at 30 mg L^−1^ had no significant differences in survival percentage, number of leaves and number of roots compared to control treatment. The percentage of increase in plant length, number of leaves, number of roots, root length, and fresh weight from in vitro rooting to acclimatization, was 40, 11, 15.6, 22 and 56%, respectively. Moreover, copper sulphate at 30 mg L^−1^ recorded the highest significant values for plant length, root length, leaf area and fresh weight (23.83 cm, 12.67 cm, 59.23 cm^2^ and 5.67 g, respectively) of acclimatized banana plants.

### 2.5. Copper Sulphate and Chemical Analyses

#### 2.5.1. Copper Sulphate and Mineral Content of In Vitro Plantlets

The mineral content of some nutrients was measured in two different growth stages of banana; in vitro rooted plantlets and acclimatized plants after 4 weeks (Table 4 and Table 5). The applied copper to the rooting medium enhanced the uptake of the studied nutrients (Ca, K, Mg, Fe, Mn and Zn) but Cu uptake increased as copper concentration increased. The highest uptakes of K, Ca, Mg, Mn and Zn (0.548, 1.253, 0.249%, 398 and 103.3 mg kg^−1^, respectively) were achieved in plants which had been treated with 60 mg L^−1^. On the other hand, 120 mg L^−1^ of copper sulphate treatment recorded the highest significant content of Cu and Fe (44.69 and 394 mg kg^−1^, respectively (Table 6). This may explain the role of copper in the growth of plantlets during the rooting stage of banana as a main component/or as an activator of some enzymes in plants [35]. This also reflects the role of Cu in many plant biophysiological processes through the high uptake rate of Fe-nutrient (due to the participation in Fe-mobilization); nutrients of K, Ca, and Mg (for the photosynthetic process); protein trafficking; and the metabolism of the cell wall [36,37,38]. At the acclimatized plant stage, the uptake rates of the previously mentioned elements decreased with the increase in copper sulphate levels in the medium up to 120 mg L^−1^ except Cu (Table 5). This decline in the nutrient uptake rate at this stage may reflect the need for plants to redistribute these nutrients in plant cells, based on the physiological activity. The previous result confirms that Cu has a dual impact (hermetic effect) in plants, including a positive impact at optimum or low levels and a toxic impact at high levels due to its high redox properties [35]. For in vitro rooted plantlets, the nutrients can be ordered as follows: Ca > K > P > Mg for macronutrients, and Mn > Fe > Zn > Cu for micronutrients. The previous order of nutrients for acclimatized plants was changed to K > Mg > Ca > P > Fe > Mn or Zn > Cu, due to the composition of the substrate used in acclimatization.

#### 2.5.2. Enzymatic Antioxidant Activities and Electrolyte Leakage

**Figure 3:**
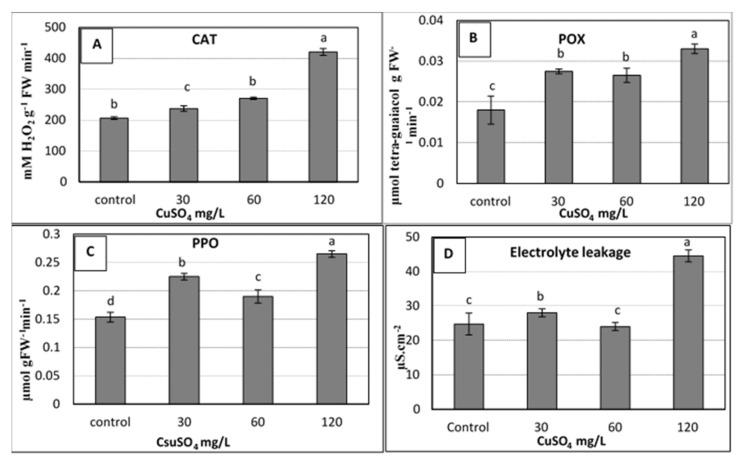
Effect of copper sulphate at 0.025, 30, 60, 120 mg L^−1^ on the activity of antioxidant enzymes: [(**A**) Catalase; (**B**) Peroxidases; (**C**) Polyphenol oxidase] and (**D**) Electrolyte leakage of banana shoots cultured in rooting medium (1 mg L^−1^ IBA) after 4 weeks of culture. Data represent the mean with SE. Letters above each bar shown the significant differences among the tested treatment, if letters were same corresponding treatments are statistically equal and vice versa according to Duncan’s multiple range tests at *p* < 0.05. Control = 0.025 mg L^−1^ and represents the concentration of CuSO_4_ 5H_2_O in MS medium (without copper sulphate supplementation).

Significant increases due to copper treatments were shown in the activity of all the studied enzymes, including catalase (CAT), peroxidases (POX), and polyphenol oxidase (PPO). The 60 and 120 mg L^−1^ copper sulphate treatments produced the highest values of activity in the studied enzymes compared to the control (Figure 3a–c). The obtained results showed that the used levels of CuSO_4_ 5H_2_O up to 120 mg L^−1^ caused an increase in the activity of the studied antioxidant enzymes (CAT, POX and PPO) due to oxidative stress that resulted from high Cu doses, which were harmful to plant cells [5]. Under this stress, plants usually stimulated the antioxidant enzyme activities that occur as a consequence of elevated levels of reactive oxygen species (ROS) such as superoxide and hydrogen peroxide [9,39]. These antioxidant enzymes, which mainly include CAT, superoxide dismutase (SOD) and POX, could alleviate the increased effect of ROS by scavenging them to harmless products [9,40].

The same trend of antioxidants was observed for electrolyte leakage (EL), for which the highest significant value of EL (~ 45 µS cm^−2^) was recorded for the highest dose of copper (120 mg L^−1^), as shown in Figure 3d. Due to its importance as an indicator for stress, the measuring of EL was used to evaluate the injuries in the cell membranes as a response to different stresses. In the current study, an increase in EL was observed when the applied copper sulphate was increased to 120 mg L^−1^ (Figure 3d). This could be due to the increase of enzymatic antioxidants, which might correlate with the high levels of ROS. These ROS have the ability to damage the nucleic acids, cause the denaturation of proteins and lipids, and, ultimately, the death of the cells [5]. Many studies confirmed the increase in EL due to stresses on banana plants such as salinity, drought stress [41], and the stress of copper sulphate as presented in the current study, whereas other studies reported that EL decreased due to calcium nitrate (0.5 to 1.0 g L^–1^) in banana plantlets [42].

#### 2.5.3. Copper Sulphate and Chlorophyll Content of In Vitro Plantlets

**Figure 4:**
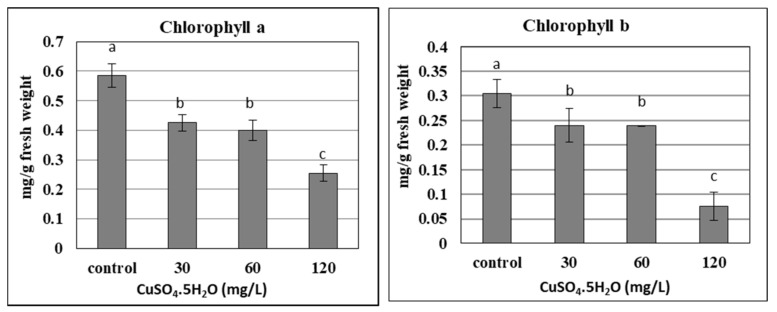
Effect of copper sulphate at 0.025, 30, 60, and 120 mg L^−1^ on chlorophyll content of in vitro banana plantlets. Data represent the mean with SE. Letters above each bar shown the significant differences among the tested treatment, if letters were same corresponding treatments are statistically equal and vice versa according to Duncan’s multiple range tests at *p* < 0.05. Control = 0.025 mg L^−1^ and represents the concentration of CuSO_4_ 5H_2_O in MS medium (without copper sulphate supplementation).

The chlorophyll content in plants is considered one of the most important components of photosynthetic pigments, which could be damaged under different stresses. The current results confirmed that the chlorophyll content (a and b) of banana plantlets decreased significantly with the increase in applied copper doses up to 120 mg L^−1^, due to the phytotoxicity of the copper (Figure 4). As the chlorophyll content was reduced due to its degradation, the photosynthesis of banana plantlets also reduced, as confirmed by many studies. Due to the stress resulting from nano silver, different concentrations led to a decrease in the chlorophyll content of banana plantlets, such as for 7 and 200 mg L^−1^ [22,23], respectively. The chlorophyll content of banana plants was significantly influenced by the liquid MS basal medium, containing excess salt (NaCl up to 800 mM) or suffering under drought stress (mannitol up to 600 mM), as reported by [41]. The stress of copper sulphate was also reported by some studies using different concentrations of up to 100 µM [17], and up to 8 mg L^−1^ [25]. The previous trend was not common for some other elements like calcium and silicon, which increased the chlorophyll content in banana plantlets when added at certain concentrations, such as with 0.5 g L^−1^ of calcium nitrate [42] and 1 g L^−1^ in the form of CaSiO_3_ [43].

### 2.6. Impacts of CuSO_4_ H_2_O Treatments on Genetic Fidelity and Genomic Template Stability

**Figure 5:**
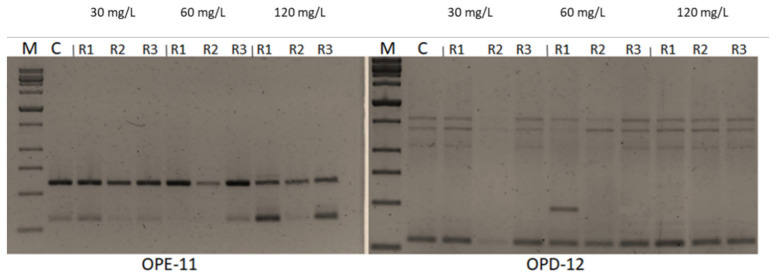
RAPD profiles of banana plants treated with 30, 60 and 120 mg L^−1^ CuSO_4_ 5H_2_O using primers OPE-11 and OPD-12. M: Molecular weight marker (1000bpSizer DNA ladder), C: Control plant. Control = 0.025 mg L^−1^ and represents the concentration of CuSO_4_ 5H_2_O in MS medium (without copper sulphate supplementation).

**Figure 6:**
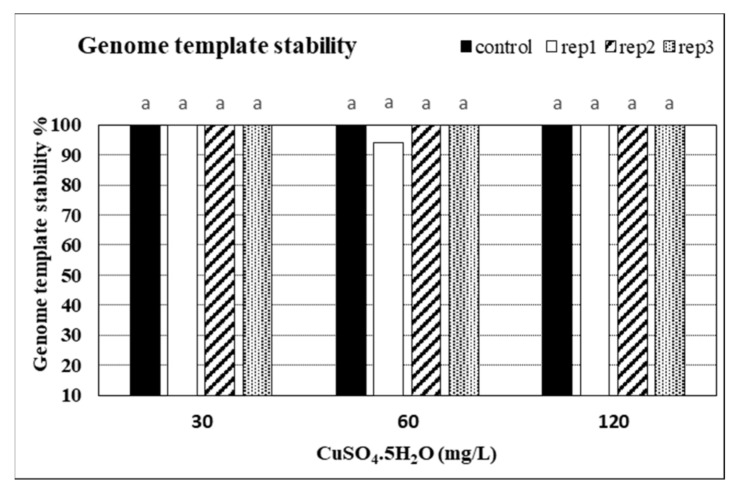
Genome template stability (GTS %) in in vitro banana plants treated with different concentrations of CuSO_4_ 5H_2_O. Control = 0.025 mg L^−1^ and represents the concentration of CuSO_4_ 5H_2_O in MS medium (without copper sulphate supplementation).

Seven -10 mer operon primers were used for screening the genome stability in response to CuSO_4_ treatments. The primers yielded specific and stable banding patterns (Table 6 and Table 7, and Figure 5). RAPD patterns generated by the copper sulphate exposed plantlets that were not clearly different from those obtained using the control DNA for all copper sulphate concentrations. The number of total bands varied from 2 (OPE-11) to 7 (OPD-14). The tested primers produced only monomorphic bands, except primer OPD-12 which produced 1 polymorphic band in the plant treated with 60 mg L^−1^ of CuSO_4_. The polymorphism percentages were 0.0% for all primers, except primer OPD-12 which gave average of 1.8%. The differences in RAPD patterns refer to the loss of normal bands and/or the appearance of new bands as compared with the control. RAPD profiles of the randomly selected in vitro plants, in comparison to the mother plant, were almost identical, thus assuring a totally genetic fidelity-maintained protocol for in vitro propagated Musa plants. Additionally, the GTS% for all treated plants was calculated (Figure 6). The GTS for control plants was fixed as 100%. There were non-significant (*p* < 0.01) differences in the GTS % of all treated plants; the average genome stability was 100, 98.9, and 100 for CuSO4 concentrations of 30, 60 and 120 mg L^−1^, respectively (Figure 5). It can be concluded from the results that the CuSO_4_ treatments did not significantly change the genome stability of in vitro *Musa* sp. plants. The RAPD method was sensitive and capable of detecting variations in plant genome profiles [44,45,46]. The RAPD technique was effectively utilized to detect genotoxic effects in several plants induced by various metals [5,47,48]. RAPD primers were used to study the genotoxic effects of CuSO_4_ for both the control and treated in vitro plantlets [49,50].

## 3. Materials and Methods

### 3.1. Plant Materials

In vitro plantlets of *Musa* sp. ‘Grand Nain’ were maintained in MS solid medium which contained 30 g L^−1^ sucrose + 2.0 g L^−1^ gelrite and was supplemented with 3 mg L^−1^ Benzyl adenine (BA) + 1 mg L^−1^ Kinetin (Kin). Cultures were kept at 25 ± 2 °C and 50 μmol m^−2^ s^−1^ photosynthetic photon flux (PPF: 16 h/d) for 3 weeks before using in this study.

### 3.2. Copper Sulphate and Bacterial Contamination (Primary Experiment)

Previously isolated endophytic bacteria as described in [5], were inoculated into MS medium containing different concentrations of copper sulphate (CuSO_4_ 5H_2_O: 0.025, 30, 60, and 120 mg L^−1^) to investigate their effect on the bacterial contamination. The medium was poured into sterilized 9 cm petri dishes and incubated for 4 days. After that they were inoculated by loop where each treatment had 4 replications. The appearance of clonal growth was used to detect the bacterial contamination of inoculated plates which were incubated at 28 °C for 3 days.

### 3.3. Copper Sulphate, Shoot Multiplication and In Vitro Rooting

Axillary shoots of about 3.0 cm were separated into clusters of three shoots each, which were used as explants. Twenty cylindrical culture jars of 375 mL capacity were used; each jar contained 60 mL MS medium, as described above, fortified with different concentrations of CuSO_4_ 5H_2_O as applied above in primary experiment (30, 60, and 120 mg L^−1^). The pH of media was adjusted to 5.8 by 0.1N KOH/HCl. The media were distributed in the culture jars and autoclaved for 20 min at 121 °C and 1.2 kg cm^−2^. MS basal medium contained 0.025 mg L^−1^ CuSO_4_ 5H_2_O which was considered a control in the current study. Cultures were sub-cultured three times, 3 weeks each on the same medium, in the same conditions as mentioned above. Contamination percentage was recorded. Then, shoot clusters (three shoots, >5 cm long) of *Musa* sp. were cultured in MS rooting medium containing 1 mg L^−1^ BA and 1 mg L^−1^ indole-3-butyric acid (IBA) and fortified with copper sulphate treatments. After four weeks of culturing on rooting medium, five replicates (jars) for each treatment, each replicate contained three explants, were selected for determination of the number of shoots, number of leaves/shoots, shoot length(cm), number of roots, root length (cm) and plantlet fresh weight (g).

### 3.4. Copper Sulphate and Acclimatization

In vitro plantlets were transplanted at the stage of 4–6 leaves into 5 cm pots filled with a mixture of sterilized peat moss and perlite (1:1). The plantlets in pots were covered with clear plastic film during the first 20 days of transplanting in air-conditioned greenhouse. The air temperature in the greenhouse was adjusted to 25 ± 2 °C, the relative humidity was 60 to 70% and the PPF to 100 µmol·m^–2^·s^–1^. After 4 weeks of acclimatization, survival percentage was calculated for the remaining plants (10 plants for each treatment at the beginning of acclimatization stage). Plant length (cm), number of leaves, number of roots, root length (cm), leaf area (cm^2^) and plant fresh weight (g) were recorded for five plants for each treatment.

### 3.5. Chemical Analyses

#### 3.5.1. Enzymatic Antioxidants and Electrolyte Leakage

Full expanded leaves of in vitro plants were used to determine the activity of some enzymatic antioxidants, e.g., catalase (CAT; EC 1.11.1.6), peroxidase (POX; EC 1.11.1.7), and polyphenol oxidase (PPO; EC 1.10.3.1), according to [50,51,52,53], respectively. Electrolyte leakage percentage was measured as described by [54,55], with some modifications according to [56] using leaf discs of in vitro plantlets. More details about the measuring of enzymatic antioxidants and electrolyte leakage can be found in [5].

#### 3.5.2. Measuring of Chlorophyll Contents

The amount of chlorophyll a (Chl a) and chlorophyll b (Chl b) in the fully expanded leaves of in vitro rooted plants was determined using spectrophotometric analysis (Double beam UV/Visible Spectrophotometer Libra S80PC, England) according to [57].

#### 3.5.3. Chemical Composition of In Vitro Plantlets

The chemical composition was carried out for oven-dried plant samples at 70 °C for 24 h. According to [58], spectrophotometer (GT 80+, UK) was used for P and the atomic absorption spectrometry (Avanta E; GBC, Canton, USA) for K, Ca, Cu, Fe, Mg, Mn, and Zn.

### 3.6. Plant DNA Extraction and RAPD-PCR Conditions

DNA from leaf material was extracted using 3 plants for each treatment by acetyltrimethyl-ammonium bromide (CTAB) according to [59]. The DNA was resuspended in distilled water and quantified by Implen P330 nanophotometer (Implen GmbH, München, Germany). Seven 10-mer primers were used for PCR amplification (Operon Technologies, USA) as presented in Table 8. PCR reactions were carried out in 20 μL volume containing: 2.0 μL of DNA (15 ng μL^−1^), 7.0 μL of dd.H2O, 10.0 μL of 10 × PCR master mix buffer, and 1.0 μL of single primer (10 pmol). The reaction mixture was subjected to the following conditions: initial denaturation at 94°C for 5 min then 35 cycles of amplification under the following parameters: template denaturation at 94 °C for 1min, primer annealing at 36 °C for 30 s, and extension at 72 °C for 3 min. By the end of the 35th cycle, final extension at 72 °C for 10 min was given, followed by storage at 4 °C. The amplification products were resolved by electrophoresis in 1.5% agarose gels in 0.5 Tris-borate-EDTA (TBE) buffer and documented on Gel Documentation system (Uvitec Cambridge Company, Cambridge, UK). The amplifications were repeated twice and only the reproducible bands were considered. Reproducible fragments were scored as ‘1’ or ‘0’ for presence or absence of the band on the gels, respectively.

### 3.7. Genomic Template Stability and Its Estimation

The polymorphic pattern was generated by RAPD-PCR profiles by using the selected primers, allowing the calculation of Genomic Template Stability (GTS %) as follows:

GTS (%) = (1 − a/n) × 100

where a is the average number of polymorphic bands detected in plants treated with different concentrations of copper sulphate and n is the number of total bands in the non-treated plants. Polymorphisms in RAPD profiles included appearance of a new band and disappearance of a band compared to the control profile. Changes in these values were calculated as a percentage of their control to compare the sensitivity of genomic template stability.

### 3.8. Statistical Analyses

All experiments under the current study were set up in a completely randomized design. Data were subjected to analysis of variance using SPSS software (version 20; IBM Corp., Armonk, NY, USA). The mean separations were performed using Duncan’s multiple range testing method and significance was determined at *p* ≤ 0.05. Deviation to the mean was calculated as Standard Error (SE). The normality of the data series was carried out by Kolmogorov–Smirnov test. When data or their transformations arcsine(x) functions) had a normal distribution, the differences in the parameter measurements due to Cu-Sulfate concentrations were analyzed by one-way analysis of variance (ANOVA). If data series did not have a normal distribution or homogeneity of variance after transformation, we evaluated response differences using a non-parametric Kruskal–Wallis H-test and a Mann–Whitney U post hoc test.

## 4. Conclusions

For the successful and efficient micropropagation protocol of banana plant, adding copper sulphate as external supplementations to culture media at the suitable concentration for each stage of in vitro propagation, could be recommended. Supplementing establishment media with 60 mg L^−1^ copper sulphate could be recommended to eliminate bacterial contamination in banana in vitro cultures with the aim of obtaining aseptic banana cultures. Banana shoots did not require the addition of external copper sulphate to multiplication media, whereas the standard content of Cu in the medium was optimal for shooting. However, copper sulphate at a level of 30 mg L^−1^ should be added to rooting media to enhance the rooting potential of micropropagated banana shoots and the acclimatization of in vitro plants. These current findings may help us to develop an applicable and cost-effective micropropagation protocol for banana plants. Further studies should be carried out to highlight the optimal concentration of Cu for in vitro cultures of uninfected explants. Moreover, future investigations should be conducted to examine the impact of the supplementation of culture media with micronutrients, rather than copper sulphate in different concentrations, on the efficiency of the micropropagation protocol of plants.

## Figures and Tables

**Table 1:** Effect of different concentrations of copper sulphate on contamination % and shoot multiplication of banana plant after three subcultures, 3 weeks each in MS medium supplemented with 3 mg L^−1^ BA + 1 mg L^−1^ Kin.

Treatments (mg L^−1^)	^1^ Contamination (%)	^2^ Number of Shoots	^2^ Number of Leaves/Shoots	^2^ Shoot Length (cm)
Control	70.0 a ± 0.176	10 a ± 0.192	4.0 ± 0.176	6.0 a ± 0.311
30	5.0 b ± 0.269	11 a ± 0.101	4.0 ± 0.117	5.7 a ± 0.294
60	0.0 c	8 b ± 0.151	3.0 ± 0.212	4.0 b ± 0.176
120	0.0 c	4 c ± 0.135	3.0 ± 0.117	2.5 b ± 0.173
Significance	*	**	NS	**

Each treatment was the mean of five replicates (jars) and each replicate contained one explant, where explant was a cluster of three shoots, except for contamination % of each treatment, which was 20 jars. Values are the mean ± SE and values followed by the same letters in the same column were not significantly different, by ^1^ Kruskal–Wallis Test and ^2^ Duncan’s test at 0.05 level. * means Significant. ** means high significant. NS means not significant at *p* ≤ 0.05. Control = 0.025 mg L^−1^ and represents the concentration of CuSO_4_ 5H_2_O in MS medium (without copper sulphate supplementation). MS medium means Murashige and Skoog medium (1962) [26].

**Table 2:** Effect of different concentrations of copper sulphate on in vitro rooting of banana plants after 4 weeks of culturing in MS medium fortified with 1 mg L^−1^ IBA.

Treatments (mg L^−1^)	^2^ Plantlet Length (cm)	^2^ Fresh Weight (g)	^2^ Number of Leaves	^1^ Roots Number	^1^ Root Length (cm)
Control	10.40 b ± 0.203	1.60 b ± 0.117	5.80 a ± 0.046	3.40 ab ± 0.058	7.78 ab ±0.196
30	14.26 a ± 0.152	2.47a ± 0.058	6.00 a ± 0.188	3.80 a ± 0.091	9.92 a ±0.135
60	11.92 b ± 0.367	1.38c ± 0.070	4.40 b ± 0.128	3.80 a ± 0.088	7.75 ab ± 0.155
120	10.28 b ± 0.164	0.61d ± 0.066	4.20 b ± 0.119	2.20 b ± 0.155	3.05 b ± 0.052
Significance	*	**	**	*	*

Each treatment was the mean of five replicates (jars) and each replicate contained one shoot. Values are the mean ± SE and values followed by the same letters in the same column were not significantly different by ^1^ Kruskal–Wallis Test and ^2^ Duncan’s test at 0.05 level. * means Significant, ** means high significant. NS means not significant at *p* ≤ 0.05. Control = 0.025 mg L^−1^ represents the concentration of CuSO_4_ 5H_2_O in MS medium (without copper sulphate supplementation).

**Table 3:** Effect of different concentrations of copper sulphate added in multiplication and rooting stages on survival and growth parameters of banana plant after 4 weeks of acclimatization.

Treatments (mg L^−1^)	^1^ Survival Percent (%)	^2^ Plant Length (cm)	^2^ Number of Leaves	^2^ Number of Roots	^2^ Root Length (cm)	^1^ Leaf Area (cm^2^)	^2^ Fresh Weight (g)
Control	100 a (±0.470)	21.55 b (±0.421)	6.00 ab (±0.529)	5.33 a (±0.208)	10.75 b (±0.327)	46.06 a (±0.317)	3.28 b (±0.225)
30	100 a (±0.588)	23.83 a (±0.503)	6.75 a (±0.123)	4.50 a (±0.257)	12.67 a (±0.241)	59.23 a (±0.169)	5.67 a (±0.200)
60	70 ab (±0.203)	14.75 c (±0.500)	5.25 c (±0.269)	2.75 b (±0.306)	8.00 c (±0.294)	35.42 ab (±0.268)	1.84 c (±0.205)
120	50 b (±0.561)	12.00 d (±0.212)	4.33 d (±0.172)	2.33 b (±0.195)	7.50 c (±0.156)	32.50 b (±0.094)	0.71 c (±0.150)
Significance	*	**	**	**	**	*	**

Each treatment was the mean of five replicates (pots) and each replicate contained one rooted plant, except survival percentage which was calculated the percentage of 10 plants (pots). Values are the mean ± SE and values followed by the same letters in the same column were not significantly different by ^1^ Kruskal–Wallis Test and ^2^ Duncan’s test at 0.05 level. * means Significant, ** means high significant, NS means not significant at *p* ≤ 0.05. Control = 0.025 mg L^−1^ represented the concentration of CuSO_4_ 5H_2_O in MS medium (without copper sulphate supplementation).

**Table 4:** Effect of different concentrations of copper sulphate on mineral content of banana in vitro rooted plants after 4 weeks.

Treatments (mg L^−1^)	^1^ K (%)	^1^ Ca (%)	^2^ P (%)	^1^ Mg (%)	^1^ Fe (mg kg^−1^)	^2^ Mn (mg kg^−1^)	^2^ Cu (mg kg^−1^)	^2^ Zn (mg kg^−1^)
Control	0.205 ab (±0.003)	0.701 b (±0.06)	0.165 d (±0.004)	0.089 c (±0.006)	229 b (±0.588)	125 d (±2.941)	1.38 d (±0.088)	44.1 d (±0.235)
30	0.142 b (±0.004)	0.832 ab (±0.005)	0.305 a (±0.003)	0.104 bc (±0.003)	220 b (±2.941)	212 c (±0.1.176)	14.88 c (±0.285)	72.5 b (±0.294)
60	0.548 a (±0.014)	1.253 a (±0.055)	0.231 c (±0.022)	0.249 a (±0.029)	341 a (±2.352)	398 a (±2.695)	23.97 b (±0.203)	103.3 a (±0.411)
120	0.244 ab (±0.049)	0.638 b (±0.023)	0.288 b (±0.024)	0.128 ab (±0.004)	394 a (±2.695)	315 b (±2.941)	44.69 a (±0.411)	64.6 c (±0.459)

Values are the mean ± SE and values followed by the same letters in the same column were not significantly different by ^1^ Kruskal–Wallis Test and ^2^ Duncan’s test at 0.05 level Control = 0.025 mg L^−1^ and represents the concentration of CuSO_4_ 5H_2_O in MS medium (without copper sulphate supplementation).

**Table 5:** Effect of different concentrations of copper sulphate on mineral content of acclimatized banana plants after 4 weeks.

Treatments (mg L^−1^)	^1^ K (%)	^1^ Ca (%)	^2^ P (%)	^2^ Mg (%)	^2^ Fe (mg kg^−1^)	^2^ Mn (mg kg^−1^)	^2^ Cu (mg kg^−1^)	^1^ Zn (mg kg^−1^)
Control	3.52 ab (±0.011)	0.290 a (±0.002)	0.138 c (±0.001)	0.382 a (±0.004)	293 a (±1.018)	153 b (±1.556)	1.70 c (±0.042)	124 bc (±0.481)
30	3.82 a (± 0.071)	0.269 ab (±0.001)	0.169 a (±0.005)	0.359 b (±0.009)	204 c (±1.176)	169 a (±1.176)	0.23 d (±0.013)	169 a (±0.444)
60	3.63 a (±0.069)	0.255 bc (±0.003)	0.150 b (±0.002)	0.272 c (±0.01)	245 b (±0.588)	136 c (±1.060)	3.14 b (±0.012)	126 ab (±0.561)
120	0.160 b (±0.018)	0.169 c (±0.002)	0.133 d (±0.003)	0.169 d (±0.003)	169 d (±1.176)	126 d (±1.020)	5.33 a (±0.051)	116 c (±0.444)

Values are the mean ± SE and values followed by the same letters in the same column were not significantly different by ^1^ Kruskal–Wallis Test and ^2^ Duncan’s test at 0.05 level. Control = 0.025 mg L^−1^ and represents the concentration of CuSO_4_ 5H_2_O in MS medium (without copper sulphate supplementation).

**Table 6:** Sequences of the selected primers used in RAPD analysis and the number of generated RAPD markers and average polymorphism percentages.

Primers	Sequence (5′→3′)	CuSO_4_ mg L^−1^	No. of RAPD Markers			No. of Polymorphic Bands	Average of Polymorphism%
			R1	R2	R3		
OPE-11	GAGTCTCAGG	30	2	2	2	0	0
		60	2	2	2	0	
		120	2	2	2	0	
OPD-12	CACCGTATCC	30	5	5	5	0	1.8
		60	6	5	5	1	
		120	5	5	5	0	
OPD-14	CTTCCCCAAG	30	7	7	7	0	0
		60	7	7	7	0	
		120	7	7	7	0	
OPH-01	GGTCGGAGAA	30	3	3	3	0	0
		60	3	3	3	0	
		120	3	3	3	0	
OPD-08	GTGTGCCCCA	30	3	3	3	0	0
		60	3	3	3	0	
		120	3	3	3	0	
OPK-08	GAACACTGGG	30	4	4	4	0	0
		60	4	4	4	0	
		120	4	4	4	0	
OPE-12	TTATCGCCCC	30	6	6	6	0	0
		60	6	6	6	0	
		120	6	6	6	0	

**Table 7:** RAPD band patterns generated from genomic DNA of copper sulphate-treated and control banana in vitro plants.

CuSO_4_ mg L^−1^	Replicates	Band Profile	Primers							Total Bands	a + b
			OPD12	OPD14	OPH01	OPD08	OPK08	OPE11	OPE12		
Control		-	5	7	3	3	4	2	6	30	-
30	1	a	0	0	0	0	0	0	0	0	0
		b	0	0	0	0	0	0	0	0	
	2	a	0	0	0	0	0	0	0	0	0
		b	0	0	0	0	0	0	0	0	
	3	a	0	0	0	0	0	0	0	0	0
		b	0	0	0	0	0	0	0	0	
60	1	a	1	0	0	0	0	0	0	1	1
		b	0	0	0	0	0	0	0	0	
	2	a	0	0	0	0	0	0	0	0	0
		b	0	0	0	0	0	0	0	0	
	3	a	0	0	0	0	0	0	0	0	0
		b	0	0	0	0	0	0	0	0	
120	1	a	0	0	0	0	0	0	0	0	0
		b	0	0	0	0	0	0	0	0	
	2	a	0	0	0	0	0	0	0	0	0
		b	0	0	0	0	0	0	0	0	
	3	a	0	0	0	0	0	0	0	0	0
		b	0	0	0	0	0	0	0	0	
Total No. of bands per primer			6	7	3	3	4	2	6		

**Table 8:** The name of primers and their nucleotide sequences.

The Name of Primer	Sequence Detail (5′→3′)
OPE-11	GAGTCTCAGG
OPD-12	CACCGTATCC
OPD-14	CTTCCCCAAG
OPH-01	GGTCGGAGAA
OPD-08	GTGTGCCCCA
OPK-08	GAACACTGGG
OPE-12	TTATCGCCCC
